# Magnolol extends lifespan and improves age-related neurodegeneration in *Caenorhabditis elegans* via increase of stress resistance

**DOI:** 10.1038/s41598-024-53374-9

**Published:** 2024-02-07

**Authors:** Jing Yu, Xiaoyan Gao, Lijun Zhang, Hang Shi, Yingxuan Yan, Yongli Han, Chengyuan Wu, Ying Liu, Minglv Fang, Cheng Huang, Shengjie Fan

**Affiliations:** School of Pharmacy, Shanghai University of Traditional Chinese Medicine, Shanghai, 201203 China

**Keywords:** Diseases, Medical research

## Abstract

Magnolol is a naturally occurring polyphenolic compound in many edible plants, which has various biological effects including anti-aging and alleviating neurodegenerative diseases. However, the underlying mechanism on longevity is uncertain. In this study, we investigated the effect of magnolol on the lifespan of *Caenorhabditis elegans* and explored the mechanism. The results showed that magnolol treatment significantly extended the  lifespan of nematode and alleviated senescence-related decline in the nematode model. Meanwhile, magnolol enhanced stress resistance to heat shock, hydrogen peroxide (H_2_O_2_), mercuric potassium chloride (MeHgCl) and paraquat (PQ) in nematode. In addition, magnolol reduced reactive oxygen species and malondialdehyde (MDA) levels, and increased superoxide dismutase and catalase (CAT) activities in nematodes. Magnolol also up-regulated gene expression of *sod-3*, *hsp16.2*, *ctl-3*, *daf-16*, *skn-1*, *hsf-1*, *sir2.1*, etc., down-regulated gene expression of *daf-2*, and promoted intranuclear translocation of daf-16 in nematodes. The lifespan-extending effect of magnolol were reversed in insulin/IGF signaling (IIS) pathway-related mutant lines, including *daf-2*, *age-1*, *daf-16*, *skn-1*, *hsf-1* and *sir-2.1*, suggesting that IIS signaling is involved in the modulation of longevity by magnolol. Furthermore, magnolol improved the age-related neurodegeneration in PD and AD *C. elegans* models. These results indicate that magnolol may enhance lifespan and health span through IIS and *sir-2.1* pathways. Thus, the current findings implicate magnolol as a potential candidate to ameliorate the symptoms of aging.

## Introduction

Aging is characterized by a gradual decline in biological function over time, leading to reduced resistance to many forms of stress, as well as increased susceptibility to a wide range of diseases^1^. Aging and age-related diseases have become a major problem that poses enormous socioeconomic and medical challenges^2^. Recently, increasing evidence has shown that the resistance to multiple forms of stress was increased in long-lived animals. For example, the long-lived Ames pygmy mouse increased resistance to external stressors such as paraquat^3^. Long-lived *daf-2* mutant *C. elegans* increased resistance to heat stress oxidative stress^4^. *Drosophila melanogaster* mutant strain methuselah (mth) increased resistance to starvation, heat and paraquat stress^5^. At present, several drugs have been reported to enhance the resistance to stress to prolong the lifespan such as nomilin, tangeretin, apple and blueberry extract^6–9^. It demonstrates that enhancing the stress resistance of an organism may be a potential anti-aging therapy.

Aging studies have been conducting using *Caenorhabditis elegans* (*C. elegans*) as a model for the highly conserved longevity genes and signaling pathways^4^. In *C. elegans*, the aging process can be regulated by signaling pathways^10,11^. Of these pathways, the preferred pathway is insulin/IGF signaling (IIS) pathway^12^. Previous studies have shown that mutants of the IIS pathway displayed lifespan-extending effects as well as a increase in the resistance to many forms of stress, including heat, H_2_O_2_, PQ, cadmium, etc.^13–15^. Besides this, IIS pathway plays a key role in the regulation of nematode growth, development, metabolism and senescence^16,17^. The IIS pathway is activated when insulin/insulin-like peptide binds to *daf-2*, a homologue of the insulin receptor located in the membrane. Immediately thereafter, the FOXO factor leads to phosphorylation of *daf-16* and inhibition of daf-16 nuclear translocation. In turn, nuclear translocation of the *daf-16* regulates the expression of many genes that control stress response, metabolism and survival^18–20^. Heat shock factor-1 (homologue of human *HSF-1*) and *SKN-1* (homologue of human *Nrf2*) are downstream transcription factors of IIS pathway, which are closely related to resistance and senescence in *C. elegans*^21–23^. *Sir-2.1* (homologue of human *sirt1*) gene has a bi-directional role in the regulation of FOXO, which may be the key to improving stress resistance and slowing down the aging process of nematodes^24,25^.

Magnolol, is a naturally occurring polyphenolic compound derived from the bark of *Magnolia officinalis* or *Magnolia grandiflora*^26^. The pharmacological studies have revealed that magnolol has effects on antioxidant, anti-inflammatory, antimicrobial, antithrombotic/antiplatelet, anti-stress, anxiolytic, anti-Alzheimer's disease, anti-stroke, hypoglycemic, smooth muscle relaxant, body weight control, anti-dyspeptic/promoter, anti-epileptic and hepatoprotective^27^. In view of its well-known antioxidant and anti-inflammatory properties, magnolol-treated organisms usually exhibit a significant reduced peroxide species, while the activity of antioxidant enzymes, such as SOD, is upregulated^28^. Despite previous findings suggesting potential anti-aging effects of magnolol, there is a paucity of literature on the underlying molecular mechanisms by which magnolol extends lifespan and health lifespan. Therefore, we used *C. elegans* as a model to explore the molecular mechanisms of magnolol on lifespan elongation.

## Materials and methods

### Reagents

Magnolol (CAS No: B21086) was obtained from Yuanye Biotech (Shanghai, China) with purity > 98%. Magnolol stock solution was prepared with dimethyl sulfoxide (DMSO) to 5 mM. The working solutions were diluted in E. Coli OP50 at final concentrations of 2.5, 5, 10, 20 and 50 μM.

### Strains and maintenance conditions

All worms were cultured at 20 °C on solid nematode growth medium (NGM) plate pre-seeded with standard food resource of Escherichia *coli* (E. *coli*) OP50^29^. The *C. elegans* strains used in this study included N2 Bristol strain (wild type), *daf-2* (*e1370*), *daf-16* (*mu86*), *DAF-16::GFP* (*muIs109*), *sir-2.1* (*ok434*), *skn-1* (*tm4241*), *hsf-1* (*ps3651*), *myo-3p::Tomm-20::mKate2::HA (FoxSi16)*, *HSP-6::GFP (zcIs13)*, *HSP-16.2::GFP (gpIs1)*, CL4176 [*smg-1 (cc546)I; dvls27 X*], NL5901 *((pkls2386)* and UA57 *(bals4).* The synchronized worm population was obtained by sodium hypochlorite treatment^30^.

### Lifespan assay

Synchronized nematodes with sodium hypochlorite solution were acquired as previously reported^31^. Synchronized L4 stage nematodes (day 0 for lifespan assays) were transferred to drug-containing and blank (DMSO only) cultures containing four different concentrations of magnolol (2.5, 5, 10 and 25 μM), 35 nematodes per group. The nematodes were transferred to the refreshed plate for 5 consecutive days until reproduction ceased, and then transferred every other day. The number of nematodes was recorded as surviving and dead during the experiment and continued until the last nematode died. The nematode death was judged by the absence of response after 10 s of touching the nematode with a platinum wire.

### Bacterial growth assay

Bacterial growth assays were performed as outlined previously^32,33^. OP50 was plotted onto solid LB medium and incubated overnight at 37 °C in inverted position. The following day, a colony was selected and inoculated into liquid LB medium and then incubated at 37 °C for 16 h. 2.5, 5, 10, 25, and 50 μM of magnolol were added to the cultured OP50. Then the samples were placed on a shaker and incubated at 37 °C for 12 h. OD595 values were measured at four different time points (0, 4, 8 and 12 h). The experiments were repeated three times independently and the statistical significance of growth inhibition was assessed by multiple t-tests.

### Chemotaxis assay

To assess the preference of nematodes for magnolol and DMSO (100%), chemotaxis assay was performed. Six small circles equidistant from the center of the circle were first drawn on an NGM plate with a diameter of 6 cm. Subsequently, 20 μl of OP50 bacterial solution (0, 2.5, 5, 10, 25 and 50 μM) containing different concentrations of magnolol was taken and added to each of the six small circles. Following immediately, approximately 50 nematodes (late L4) were transferred to the center of the circles of the NGM plates. 2 hours later, the number of nematodes crawling onto each lawn was counted.

### Progeny production assay

Ten synchronized wild-type L4-stage nematodes were transferred to NMG plates with or without magnolol (one nematode per plate) to count the number of progeny that hatched. Nematodes were transferred to a new dish every 24 h until spawning ceased. All plates continued to be incubated in a 20 °C incubator to record the number of eggs hatched. The experiment was repeated three times.

### Locomation and pumping rate assays

Synchronized worms were cultured as lifespan analysis, and locomotion and pumping rate were recorded as previous methods^34^.

#### Pharyngeal pumping assay

At L4 larval stage, (n = 3 experiments, 10 nematodes per group) nematodes were treated with DMSO and a concentration of Magnolol (5 µM), respectively. Age-synchronized larval cultures were performed as described in Section "Lifespan assay". On days 3, 5, 7, 9, 11, 13, and 15 of the adulthood, pharyngeal contractions were observed under a light microscope to calculate the 30-s pharyngeal pumping rate.

#### Locomotion assay

Similarly, to evaluate the motility of nematodes, nematodes were transferred to a new NGM on days 3, 5, 7, 9, 11, 13, and 15 of the adulthood, and the number of sinusoidal curves formed by nematodes within 30 s was observed under an optical microscope. In addition to this, we also rated the nematodes' locomotor ability, according to their locomotor state, we classified their locomotor behavior into three levels, A (energetic, symmetrical and spontaneous movements), B (rigid, uncoordinated movements that often must be stimulated) and C (moving only the head or tail under stimulation). The levels of locomotor behavior were recorded on days 9, 11, 13 and 15 of the adulthood.

### Oil red O staining

Nematodes were treated with magnolol (5 µM) from L1 to L4 period. The nematodes were washed down to a 1.5 ml centrifuge tube with S Buffer and repeatedly washed 5 times. Discard the S Buffer to harvest clean nematodes. Nematodes were immobilized for 15 min by adding 500 µl of 60% isopropanol to the centrifuge tube. Subsequently, 60% isopropanol was discarded and 500 µl of 0.3 g/L oil red working solution was added and incubated on shaker for 5 h for staining. Immediately after washing with M9 buffer, it was placed on a 2% agarose gel pad. Nematodes were observed under a German Leica laser scanning confocal microscope. The average intensity of oil red O staining was quantified using ImageJ.

### Mitochondrial integrity analysis

The *myo-3p::Tomm-20::mKate2::HA* (*FoxSi16*) nematodes were synchronized and inoculated into MGM containing DMSO and magnolol, respectively. The nematodes were then transferred to slides coated with a 3% agarose pad and then anesthetized with 0.5% NaN3. The integrity of nematode *myo-3p::Tomm-20::mKate2::HA* (*FoxSi16*) mitochondria was observed under a German Leica laser scanning confocal microscope. At least ten nematodes were observed per group. The fluorescence intensity of somatic mitochondria was characterized using ImageJ software.

### Aging-related neurodegenerative disease assays

The UA57 strain and the wild-type N2 strain were synchronized and grown to the L1 stage at 20 °C. Then the nematodes containing approximately 400 L1 stage nematodes were transferred to a new 1.5 ml EP tube, centrifuged (3500 rpm, 3 min), and the supernatant discarded. Immediately afterward, the worms were washed three times with M9. After resuspension, the drug was added and incubated for 30 min, then 6-OHDA (10 mM) was added and incubated for 1 h avoiding light to induce dopaminergic neuron death. After the treatment, the worms were washed three times with M9 and transferred to new NGM plates containing the drug. Blank control and positive control groups were done the same treatment with DMSO and cabergoline (Caber) respectively. After 9 days of drug treatment, the nematodes were transferred to NGM plates with OP50 and after 10 s of acclimatization, the genus showed sinusoidal movement of the nematodes in 30 s. The worms were then transferred to 2% agarose gel pads with a platinum wire and then anesthetized with 0.5% NaN3. The nematodes were observed under a German Leica laser scanning confocal microscope. Fluorescence intensity of nematode heads was quantified using ImageJ. For the experiment of detecting α-synuclein in the muscle tissue of the body wall of NL5901 nematode, NL5901 strain was synchronized and grew to L1 stage at 20 °C. The nematodes were then transferred to a new drug-containing NGM. Seven days after dosing, the worms were transferred to 2% agarose gel pads with platinum wires and then anesthetized with 0.5% NaN3. Nematodes were observed under a German Leica laser scanning confocal microscope. The fluorescence intensity of nematode head was quantified by ImageJ. Blank and positive controls were treated identically with DMSO and cabergoline, respectively.

Strain *CL4176* (A-β transgenic in muscle cells) was synchronized and incubated at 16 °C for 36 h to L3 larval stage. Then the worms were transferred to a new NGM plate and incubated at 25 °C. The state of the worms was observed and recorded daily using a stereomicroscope. Worms were considered to exhibit the pathological behavior of paralysis if they could not crawl forward or backward when lightly touched with a platinum wire. The number of paralyzed worms was determined accordingly and the paralysis curve was plotted using GraphPad Prism 8.

The analysis of *skn-1* RNA interference (RNAi) was performed as described previously^35^. Briefly, RNAi bacterias were grown at 37 °C in LB liquid medium overnight with 50 μg/ml ampicillin, and then seeded onto NGM plate containing 50 μg/ml ampicillin and 1 mg/ml isopropylthiogalactoside (IPTG), cultured overnight. L1 stage NL5901 and CL4176 worms were transferred onto the plate with RNAi bacteria lawn until L4 stage. The remaining steps were carried out as described in the above.

### Stress resistance assays

#### Heat shock assay

During the heat shock assay, the nematodes of L4 period were firstly divided into magnolol group and control group, with the number of nematodes in each group around 50. Prior to exposure to the heat exciters, the nematodes in the thymol group were placed on Petri dishes containing magnolol for 5 days of incubation, while the blank control nematodes were placed on control plates containing DMSO for 5 days of incubation. After 5 d of drug treatment, the nematodes were placed on new NGM and incubated at 35 °C. The number of nematode death was recorded every two hours until all nematodes died.

#### Oxidative stress assay

In the in vivo oxidative stress assay, nematodes were treated as shown in 2.7.1 prior to exposure to the stressors. After 5 d of drug treatment, nematodes were transferred to new NGM plates containing 8 mM H_2_O_2_. Incubation was carried out at 20 °C and monitored hourly until the nematodes died out. Besides the above mentioned, the nematodes were also exposed PQ (200 μM), MeHgCl (2 μM) and colchicine (4 mM). Nematode mortality was observed every two hours until the number of nematode deaths reached the LD50 level.

The oxidative stress assay was performed using a total antioxidant capacity assay kit (Biyun Tian, China). The ABTS mixture was firstly prepared according to the instructions of Total Antioxidant Capacity Assay Kit (Biyuntian, China). Subsequently, 20 μl of peroxidase working solution was added to each assay well in 96-well plate. And then 10 μl of thujaplicin and glutathione standards were added. Finally, 170 μl of ABTS mix was added. The reaction system was incubated at room temperature for 6 min and the absorbance was detected at 414 nm (ΔA414 = A414_Blank control_ − A414_Standard/Sample_).

#### Antioxidant capacity assay

The assay of in vitro and in vivo antioxidant capacity of magnolol was established on the basis of previous studies^36^. The antioxidant capacity of nematodes was determined in vivo through the detection of reactive oxygen species (ROS) levels in nematodes measured by 2,7-dichlorodiacetate fluorescein (DCFH-DA). After nematodes were administered with magnolol (5 µM) for 4 d, about 2000 worms were collected in M9 buffer and then washed three times with M9 and placed in a − 80 °C refrigerator. Nematodes were removed from the refrigerator and sonicated for 30 s followed by centrifugation at 4 °C for 5 min. Finally, 50 µl of the supernatant was taken to measure the fluorescence intensity at emission/excitation wavelengths of 530 and 485 nm using a microplate reader (BioTek Instruments).

The contents of intracellular reactive oxygen species (ROS), malondialdehyde (MDA), activities of superoxide dismutase (SOD) and catalase (CAT) were assayed. The enzymatic activities of MDA, CAT and SOD in nematodes were detected by using CAT and SOD (Biyuntian, China) kits. Firstly, L4 stage nematodes were taken and washed with M9. Then, the nematodes were sonicated for 30 s and centrifuged at 4 °C for 5 min, after which the supernatant was extracted. Nematode proteins were extracted by the above process. The total protein in each sample was then measured by an enzyme-linked immunosorbent assay (ELISA) kit based on BCA method. Finally, the activities of MDA, CAT and SOD in each sample were measured by the instructions of the ELISA kit for MDA, CAT and SOD (Biyuntian, China).

#### DAF-16::GFP translocation experiments

Nematodes containing DAF16::GFP (n = 3 experiments, 10 animals per group) were synchronized and separately inoculated into MGM containing DMSO and magnolol, and cultured to L4 stage. The nematodes were then transferred to slides coated with 2% agarose pads, and then anesthetized with 0.5% NaN3. The nuclear translocation of nematode containing DAF-16::GFP was observed under a German Leica laser scanning confocal microscope. The number of GFP-positive nuclei of each worm was calculated.

#### Analysis of the fluorescence intensity of HSP16.2::GFP and HSP-6::GFP

Nematodes in L1 stage were treated with or without magnolol for 3 days and then incubated at 37 °C for 1 h. Nematodes were then anesthetized with a 3% agarose pad of sodium azide and images were obtained under a fluorescence microscope (Leica, Wetzlar, Germany). Image J software was used to calculate the fluorescence intensity of HSP-6:: GFP and HSP-16.2::GFP.

#### Cell cultures and cell viability assay

To evaluate the cytotoxicity of magnolol, L02 and HepG2 cells were added to 96-well plates at a concentration of 1 × 10^5^ cells per well and incubated at 37 °C. The cells were observed to be well adhered to the wall, the medium of each well was aspirated and medium containing five (0, 2.5, 5, 10, 25 and 50 μM) different concentrations of magnolol. The cells were then incubated at 37 °C for 24 h. Immediately after that, 10 μl of Cell Counting Kit 8 (CCK8) (TargetMol, Cat: C0005) reagent was added to each well and incubated at 37 °C for 2 h. The OD values were measured at 450 nm. Statistical analysis was performed using GraphPad Prism 8 software. In addition to this, we also administered L02 and HepG2 cells for 24 h by the method mentioned above. The cells were then exposed to potassium-based mercury chloride and paraquat for 24 h, and cell viability was assayed using the method described above. (Survival rate = (OD experiment − OD blank)/ (OD control − OD blank) × 100%).

### Quantitative real-time polymerase chain reaction (RT-PCR) assay

For gene expression analysis, nematodes after synchronization were transferred to NGM containing magnolol (5 μM), incubated to L4 period, and collected for quantitative realtime PCR. Briefly, total RNA was extracted from approximately 1,000 nematodes using Trizol reagent (Vazyme, Nanjing, China) according to the manufacturer's instructions. Total RNA was then used as a template for reverse transcription into complementary DNA using a cDNA kit (Vazyme, Nanjing, China) in conjunction with ABI StepOnePlus Real-Time Polymerase Chain Reaction System (Applied Biosystems, Foster City, CA, USA). This was followed by RT-qPCR using SYBR Green PCR Master Mix (Shanghai Sangon Biotechnology, China). β-actin was used as the internal reference for the expression level of mRNA of all genes. Statistical analysis was carried out by using 2^−∆∆Ct^ method. The sequences of all primers were listed in Table 1.

**Table 1:** List of primers for quantitative real-time PCR in *C. elegans.*

Gene	Forward primer	Reverse primer
*β-actin*	TCGGTATGGGACAGAAGGAC	CATCCCAGTTGGTGACGATA
*daf-2*	TCGAGCTCTTCCTACGGTGT	CATCTTGTCCACCACGTGTC
*age-1*	CCTGAACCGACTGCCAATC	GTGCTTGACGAGATATGTGTATTG
*daf-16*	TCAAGCCAATGCCACTACC	TGGAAGAGCCGATGAAGAAG
*sod-3*	AGCATCATGCCACCTACGTGA	CACCACCATTGAATTTCAGCG
*hsf-1*	TTGACGACGACAAGCTTCCAGT	AAAGCTTGCACCAGAATCATCCC
*hsp-16.2*	CTGCAGAATCTCTCCATCTGAGTC	AGATTCGAAGCAACTGCACC
*sod-2*	GCTCTTCAGCCAGCTCTC	AGTATCCCAACCATCCCC
*Sod-1*	GTGCTGTCGCTGTTCTTC	GGTCCACCATGAGTCTTTC
*cat-1*	CTTCTCGCCCTTCTTGCT	TCCGATGGTGATTGCTCC
*ctl-1*	ATACTGCTGCTTCTCGTC	TCATCCCACATCTTTTTG
*ctl-2*	AGATGTGGCGTATGTCCT	AGATGTGGCGTATGTCCT
*ctl-3*	TCAACGGTCGCTGGAGAA	TTGCGTCACGAATGAAGAAG
*mtl-1*	AAGTACTGCTGTGAGGAGGC	GTTCCCTGGTGTTGATGGGT
*mtl-2*	CGGTTGTTAATAAATACGG	AATGTTGGAAGAGGAGCT
*akt-1*	AACATGGACGCAACAAGCAC	TTCCGAAGGTTCCTTGACCG
*sir2.1*	GCAAGAAATAACGGAGGA	TTTGAGCACGACGAAGAT
*cyp-13a7*	AAAAATGGCAATGGGACAAG	AATACTTTGAATATCGGTAG
*cyp-13a11*	GCAAATTCTCGCCGTTGTAT	TCGTCTCCTGATTCCCATCT
*cyp-14a1*	CCTTTCTTGGGGTCTCATCA	AAGTAGCGGCTTGGATTGAA
*cyp-14a3*	CAGGCACTGGAGACAAATCA	CAGGCACTGGAGACAAATCA
*cyp-35a1*	CGGAGTCACTGTTGCTCAAGCC	AGACTTCAAACGCAGCACCCATG
*cyp-35a2*	ACTGGTGGCATTGTTTCGACTCTC	GGAATTGGTCCGACCCATAGTGTG
*cyp-35a3*	GCTCAACTCAGTGCTCTCCATGTC	TCCCAGGCAACTTCTCTTTCCAAC
*cyp-35a4*	CTGACCGTGCTTCAACTCCATACC	TCCAGCATCGACAGGGTGACC
*cyp-35a5*	GGGAAGGAGCCGATGGAAATCAAG	GGGAAGGAGCCGATGGAAATCAAG
*cyp-35b1*	TGAACACGAGATGTGCCGAA	AACGTTTTCCGACGAGCAGA
*cyp-35b2*	GTTCCTCCCGCCTGTTTTCT	TTTCCTCGCATCTTGCATCC
*cyp-35b3*	GTGATTATGAAACGTCGCAAGAAG	GCGGATGCTGTAAATGGAAAGAC
*cyp-35C1*	AAAGTGACTAACGGAGGATCTCG	CTAGCAAGAGCCGAGCTGTATTT
*cyp-36a1*	GGTGGAAGGCTCAACGACGATTC	GCCAACGAAGCAATTGTGTCCTG
*gst-4*	CCAAATGGAGTCGTTGGCTTC	TTTGATGCTCGTGCTCTTGC
*gst-10*	GGAGTCCGCGATGTTCGTAT	TTCACTAGAGCCTCCGGGAT
*ugt-44*	GCACATTTTGGTATGCTCTGCT	CGGCAACAGAAGGGTCACAT
*pgp-3*	GTGATGGGACTTCCTGACGG	CTTTGGGTCTCTGACAATCGC
*pgp-12*	CCACTCATGTACCACGGCAT	AATAGCATTCCAGCGGCAGT
*pgp-13*	CCGATGGCATAGACACCGAA	GCTTCTTGCACAGCCCTTTC
*pgp-14*	AGGAGTACGGTGCTAGCGAT	ACATCTTTGGGGCGTCATCA

### Statistical analysis

All tests were statistically analyzed using GraphPad Prism 8.0 (GraphPad Software Inc., San Diego, CA, USA) and SPSS (version 21.0). Lifespan test results were analyzed using Kaplan–Meier survival analysis, and between-group comparisons were scored for significance using the log-rank test. Comparison of data between the two groups was analyzed using Student's t-test. Values have been expressed as ± SEM. Statistical analysis was performed using SPSS statistical software. *p*-values less than or equal to 0.05 were considered statistically significant.

## Results

### Magnolol extends lifespan in *C. elegans*

**Figure 1:**
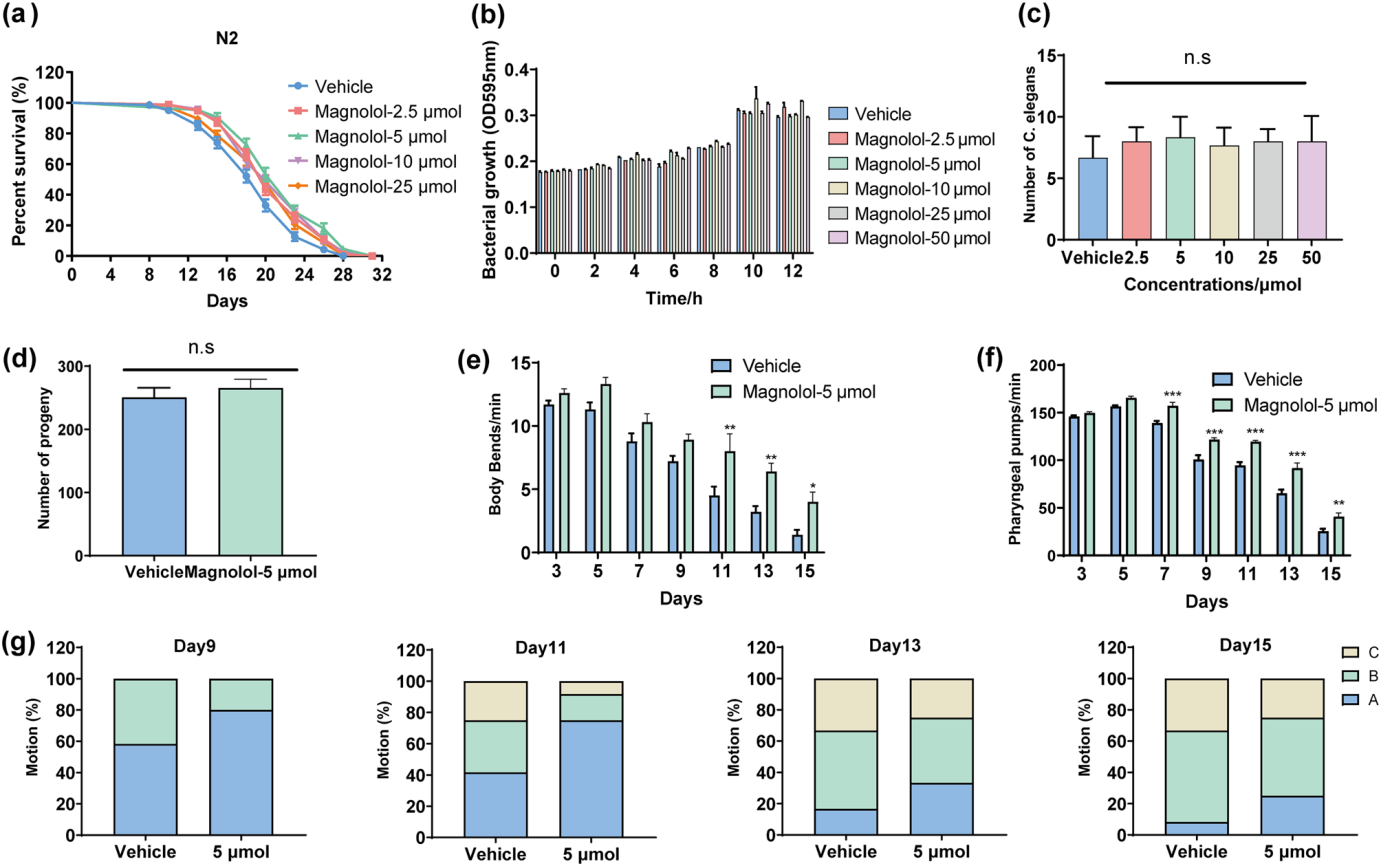
Effect of magnolol on lifespan and health lifespan in wild-type *C. elegans* (N2). (**a**) Nematodes were treated with different concentrations of magnolol at L4 stage at 20 °C. Mortality nematodes were counted every other day until all nematodes were dead (n = 3 experiments, each involving > 100 animals). The detailed lifespan data were exhibited in Table 2. (**b**) Effect of magnolol on the growth of *E. coli OP50* (results repeated three times). (**c**) Nematode "chemotaxis" towards *E. coli* OP50 containing different concentrations of magnolol (n = 3 experiments, each involving 50 animals). (**d**) Effect of magnolol on nematode reproduction. (**e**) Effect of magnolol on the frequency of body bends in nematodes. (**f**) Effect of magnolol on the frequency of the pharyngeal pumps in nematodes. (**g**) Measurement of three levels of motility. Locomotor ability was tested on days 9, 11, 13, and 15 (n = 3 experiments). Significance was analyzed by Two-tailed unpaired Student’s t-test (**d**), one-way ANOVA (**c**), and two-way ANOVA (**b**, **e**, **f**). All data were presented as mean ± S.E.M. Compared with vehicle group, *p < 0.05, **p < 0.01, ***p < 0.001.

Magnolol has been reported to exert significant anti-oxidation effects and can scavenge excess free radicals from the body, which is consistent with the free radical doctrine of aging^37^. To investigate whether magnolol could prolong lifespan, we conducted a longevity test in *C. elegans*. The result showed that the average lifespan of the control group was 19.480 ± 0.383 days. After 2.5, 5, 10 and 25 μM of magnolol interventions, the average lifespan of the worms was increased to 21.120 ± 0.330, 22.039 ± 0.400, 21.384 ± 0.376 and 20.533 ± 0.351 days (*P* < 0.05), which was 8.42, 13.14, 9.78 and 5.4% higher compared to the control group (Table 2). Interestingly, magnolol significantly prolonged the lifespan of *C. elegans* and the best lifespan extension effect was observed at a concentration of 5 μM (Fig. 1a) inferring that high concentrations of magnolol may have some unknown side-effects on nematodes.

**Table 2:** Effects of magnolol on the lifespan of N2 *C. elegans.*

Strain	Group	Number	Mean life (days)	Maximum longevity (days)	Median survival time (days)	Increase (%)	*p* value
N2	Vehicle	142	19.480 ± 0.383	28	20.00	/	/
	Magnolol-2.5 μM	175	21.120 ± 0.330	31	20.00	8.42	0.01**
	Magnolol-5 μM	128	22.039 ± 0.400	31	23.00	13.14	< 0.001***
	Magnolol-10 μM	146	21.384 ± 0.376	31	20.00	9.78	< 0.001***
	Magnolol-25 μM	184	20.533 ± 0.351	31	20.00	5.4	0.008**

Lifespan experiments were analysed using Kaplan–Meier survival analysis and compared among groups, scoring for significance using the log-rank test. All data were expressed as mean ± SEM. **p < 0.01, ***p < 0.001 versus control group.

As known, dietary restriction prolongs lifespan in nematodes which are fed with E. *coli* OP50 (the standard experimental food for nematodes). While, previous studies showed that magnolol has antimicrobial effects^9,38^. To exclude the possibility that the lifespan-extending effect of magnolol was due to a decrease in food intake, we conducted the following experiments. First, by E. *coli* OP50 growth assay, we found that magnolol did not chang the growth rate of E. *coli* OP50 (Fig. 1b). Second, a chemotaxis assay revealed that nematodes had no dietary preference for OP50 containing excipients DMSO and magnolol (Fig. 1c), suggesting that effects of magnolol were not due to inhibition of food intake. In addition, the "disposable soma" theory of aging suggests that inhibition of reproduction may prolong worm lifespan. To investigate whether magnolol affects nematode reproduction, we examined the average number of hatchlings and the number of larval progeny (Fig. 1d). The results showed no significant difference between the magnolol treated and control groups. Thus, these results suggest that magnolol directly extends the lifespan of nematodes.

### Magnolol improves healthspan in *C. elegans*

Aging is commonly accompanied by muscle loss which in turn affects body's locomotor behaviors, such as body bending, head swinging, and pharyngeal pumping in *C elegans*. To investigate the effect of magnolol on the health lifespan of nematodes, we tested the locomotor behavioral ability of aged N2. Pharyngeal pumping and body bending rates of nematodes were calculated from day 3 to day 15 (Fig. 1e, f), which showed that magnolol significantly improved pharyngeal pumping rates from day 5 to day 15, as well as the rates of body bending from day 7 to day 15 compared to the control group. In addition, we assessed the motility of N2 which is a key indicator of muscle integrity and affects the health span (Fig. 1g). The results showed that on day 15th, more than 25% of worms treated with magnolol were classified as Class A when compared to only about 10% in the control group. These results suggest that magnolol could improve locomotor status in *C. elegans*.

### Magnolol reduces lipid accumulation in *C. elegans*

**Figure 2:**
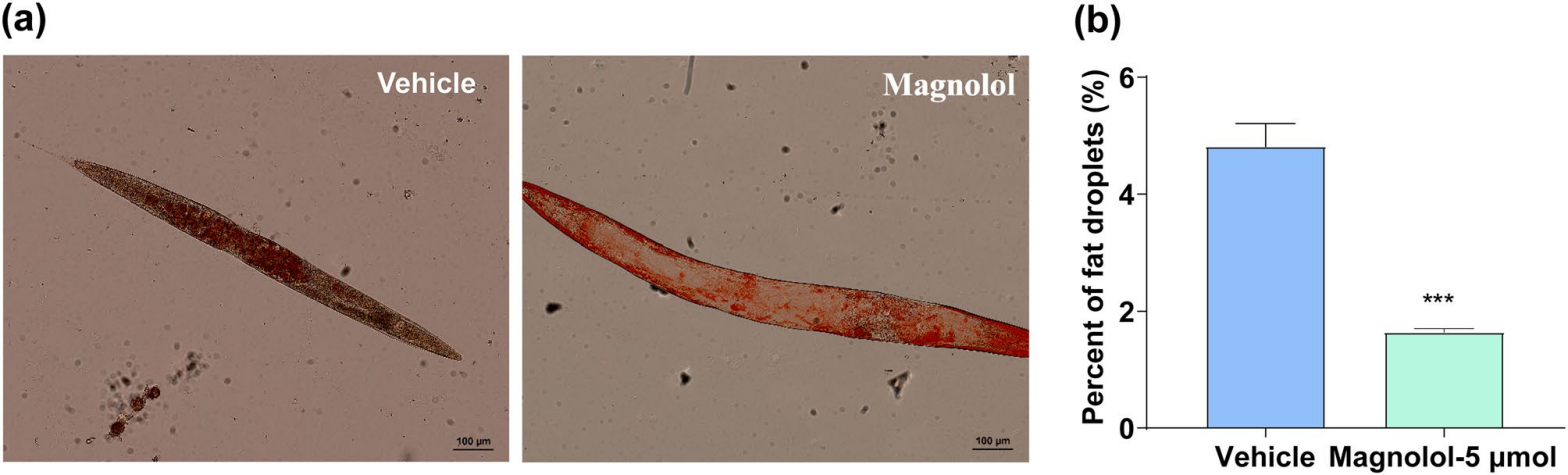
Magnolol reduces lipid accumulation in *C. elegans*. (**a**) Oil red O staining of fat accumulation in nematodes. (**b**) Statistics analysis of (**a**) (n = 3 experiments). Significance was analyzed by Two-tailed unpaired Student’s t-test. All data were presented as mean ± S.E.M. Compared with vehicle group, *p < 0.05, **p < 0.01, ***p < 0.001.

During aging process, lipids were accumulated in the body of worms, which can lead to a variety of age-related diseases^39,40^. Therefore, the regulation of fat metabolism is crucial for aging and aging-related diseases. To investigate the effect of magnolol on lipid accumulation, oil red O staining was applied to mark whole-body lipid droplets in *C. elegans*. The results showed that magnolol could significantly reduce lipid accumulation compared with the control group (Fig. 2a, b), suggesting that magnolol can regulate the metabolism of lipids in nematodes.

### Magnolol improves stress resistance and toxin resistance in *C. elegans*

**Figure 3:**
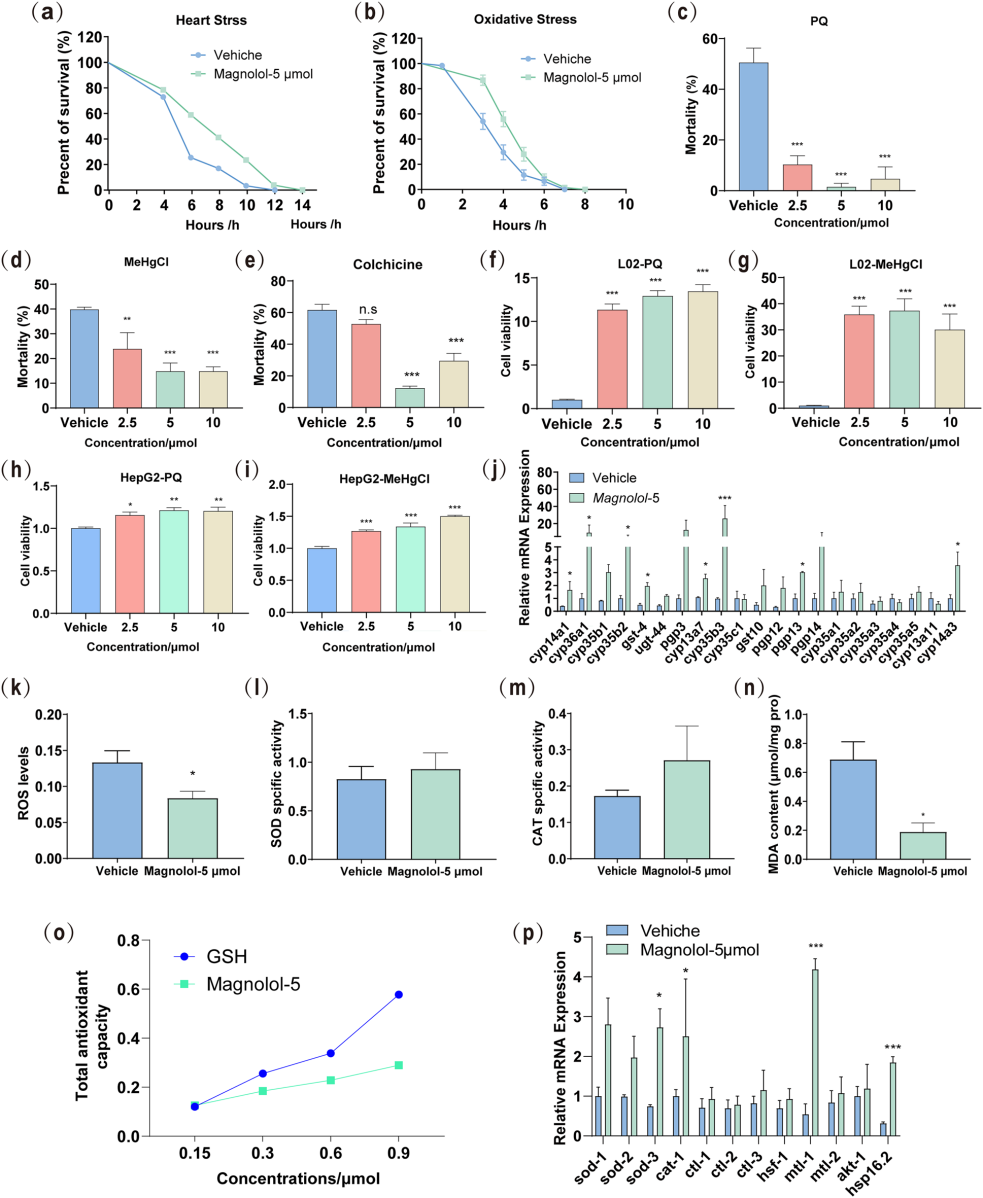
Life-extending effects of magnolol may be associated with increased stress resistance. (**a**) Survival curves of nematodes under 35 °C for heat stress analysis (n = 50–60 nematodes for each group each time, experiment repeated 3 times). (**b**) Survival curves of nematodes exposed to hydrogen peroxide (final concentration: 8 mM) for oxidative stress analysis (n = 60–70 nematodes for each group each time, experiment repeated 3 times). The survival data were analyzed by log-rank test, and the detailed data were concluded in Table 3. (**c**) The resistance to oxidative stress of N2 worms to 200 mM paraquat (PQ). About 50 worms in Magnolol. (**d**) The survival rate of N2 treated by 2 μM MeHgCl. (**e**) The livability of N2 exposed to 4 mM colchicine (CC). (**f**) The cellular viability of LO_2_ exposed to 500 μM paraquat (PQ). (g) The cellular livability of LO_2_ exposed to 2 μM MeHgCl. (**h**) The cellular viability of HepG2 exposed to 500 μM paraquat (PQ). (**i**) The cellular livability of HepG2 exposed to 2 μM MeHgCl. (**j**) qRT-PCR analysis of detoxification genes (n = 4). (k) ROS content in nematodes after seven days of magnolol treatment. (**l**) The superoxide dismutase (SOD) activity in nematodes after treatment with magnolol. (**m**) Catalase activity. (n) Levels of malondialdehyde. (**o**) Total antioxidant capacity of magnolol and glutathione (GSH). (***p***) qRT-PCR analysis of stress resistance-related genes (n = 4). Significance was analyzed by Two-tailed unpaired Student’s t-test (**d**, **e**); One-way ANOVA. Data were expressed as mean ± S.E.M. and n ≥ 3 in each experiment. Compared to vehicle group, *p < 0.05, **p < 0.01, ***p < 0.001.

Studies have shown that increased longevity and health span are usually accompanied with enhanced stress tolerance^41^. Therefore, we assayed the effect of magnolol against stressors. First, we observed whether magnolol could increase the resistance of nematodes under heat and oxidative stress conditions. For the heat stress experiment, nematodes treated with magnolol were incubated in a 35 °C incubator until death. The results showed that magnolol significantly prolonged the average lifespan of nematodes compared with the control (Fig. 3a, Table 3). For the oxidative stress experiment, we exposed nematodes to NGM plates containing 8 mM hydrogen peroxide (H_2_O_2_) and placed them in a 25 °C incubator until death. We found that the average lifespan of the nematodes treated with magnolol was significantly prolonged compared with the control group (Fig. 3b, Table 3). In addition, we also exposed adult nematodes to toxins, including PQ, MeHgCl and colchicine, and the results showed that magnolol increased toxic resistance in vivo (Fig. 3c–e). The toxic test with PQ or MeHgCl also explored in human hepatic cell lines L02 and HepG2. The results showed that magnolol improved the cell viability of both HepG2 and L02 when exposed to PQ, MeHgCl (Fig. 3f–i), suggesting that magnolol had detoxification or toxic resistance effects. In addition, RT-RCR experiments showed a significant increase in the expression of stress resistance genes *sod-3*, *cat-1*, *mtl-1*, *hsp16.2* in worms after treatment with magnolol (Fig. 3p).

**Table 3:** Effects of magnolol on the lifespan of N2 *C. elegans* under conditions of 35 °C and H_2_O_2_.

Strain	Group	Number	Mean Survival ± SEM (hours)	Maximum longevity (hours)	Median survival time (hours)	Increase (%)	*p* value
N2 (35 °C)	Vehicle	59	6.37 ± 0.28	12	6	/	/
	Magnolol-5 μM	51	7.91 ± 0.24	14	8	24.18	< 0.001***
N2 (H2O2)	Vehicle	61	3.98 ± 0.16	7	4	/	/
	Magnolol-5 μM	68	4.81 ± 0.15	8	5	20.85	< 0.001***

Lifespan experiments were analysed using Kaplan–Meier survival analysis and compared among groups, scoring for significance using the log-rank test. All data were expressed as mean ± SEM. ***p < 0.001 versus control group.

To further investigate the relationship between the lifespan-extending effect of magnolol and the anti-stress effect, we examined the effect of magnolol aganist oxidative stress in vivo and in vitro. It has been reported that the drug itself has redox properties that can reduce excess oxygen negative ions in the body, and in this way increase the antioxidant reduction capacity of the body. Therefore, we first tested the antioxidant capacity of magnolol in vitro using a ABTS cation scavenging assay (Fig. 3o), which showed that magnolol exhibited strong anti-oxidative effect similar to the positive control glutathione (GSH). Further, excessive ROS levels are responsible for aging^42^. And under stressful conditions can lead to the accumulation of ROS^43^. Therefore, the fluorescent probe H2DCFDA method was used to detect the effect of magnolol on ROS levels in vivo. ROS level was reduced in magnolol-treated group compared with that of control group in aged *C. elegans* (Fig. 3k). In addition, we also examined the expression levels of CAT, MDA and SOD in vivo treated with magnolol. The results showed that magnolol significantly reduced MDA level (Fig. 3n), and have a tendency to increase the content of CAT and SOD enzymes in nematodes (Fig. 3i, m), suggesting that magnolol is also capable of antioxidant.

As known, toxins including ROS and xenobiotics can be detoxified through a three-step procedure. Phase I detoxification enzymes, such as CYPs; chemically modify toxins and then binds to Phase II enzymes, making them more water soluble^44^; finally, these modified toxins are secreted out of the cells by ABC transporters as Phase III^45^. To understand why magnolol showed toxic resistance effect, several gene mRNA levels related detoxification were tested with qRT-PCR analysis. The result showed that Phase I enzymes *cyp13a4*, *cyp14a1*, *cyp14a3*, *cyp36a1*, *cyp35b2* and *cyp35b3*, Phase II *gst-4* and *gst-10*, and Phase III *pgp-13* were transcriptionally upregulated in worms after magnolol treatment (Fig. 3j). These results suggest that the positive regulation of magnolol on detoxification gene expression for stress resistance may be one of the key mechanisms of its anti-aging effect.

### Magnolol has a protective effect on mitochondrial morphology

**Figure 4:**
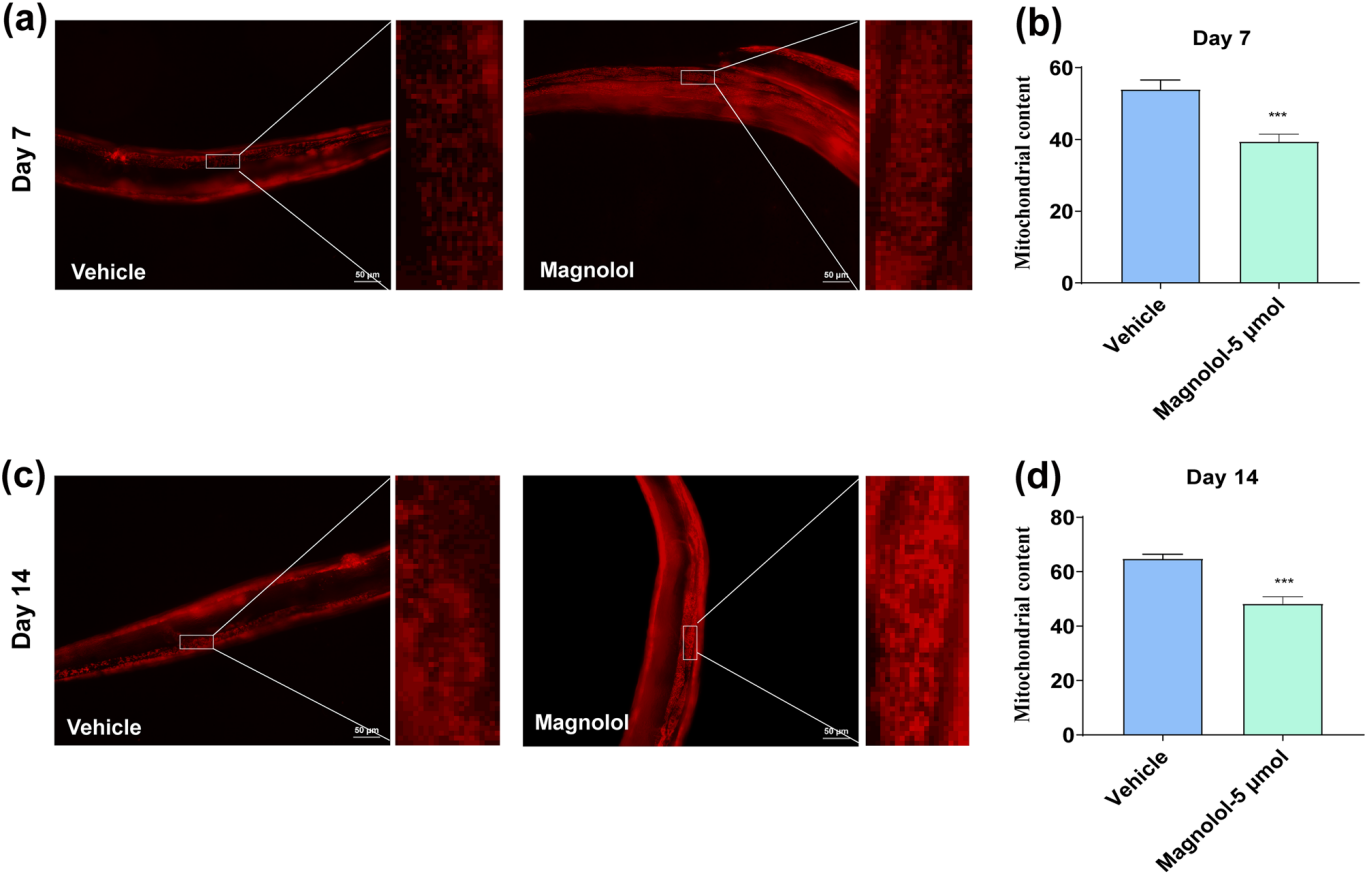
Magnolol has a protective effect on mitochondria. (**a**, **b**) Representative images and RFP quantification of mitochondrial content at day 7 of adulthood in muscle *(p*_*myo-3*_* mtRFP) mito::RFP* reporter strains (n ≥ 10). Scale bar, 50 μm. (**c**, **d**) Representative images and RFP quantification of mitochondrial content at day 14 of adulthood in muscle *(p*_*myo-3*_* mtRFP) mito::RFP* reporter strains treated with magnolol or vehicle, (n ≥ 10). Scale bar, 50 μm. The data were analyzed by Two-tailed unpaired Student’s t-test. Compared to vehicle group, ***p < 0.001.

Additional of detoxication gene regulation, mitochondrial homeostasis also plays a vital role in aging^46,47^. Mitochondrial morphology and dynamics are closely related to aging. Protection of mitochondrial homeostasis allows the body to be protected from stressful irritations, thus increasing resistance. To investigate the regulatory effect of magnolol on mitochondria, a muscle mitochondria reporter-expressed *C. elegans* line, *myo-3p::TOMM-20::mKate2::HA* (*foxSi16*), was treated with vehicle and magnolol for morphological observation of mitochondria. The results revealed that magnolol reduced mitochondrial damage due to senescence (Fig. 4a–d).

### Magnolol decreases HSP-6 and HSP-16.2 under heat stress

**Figure 5:**
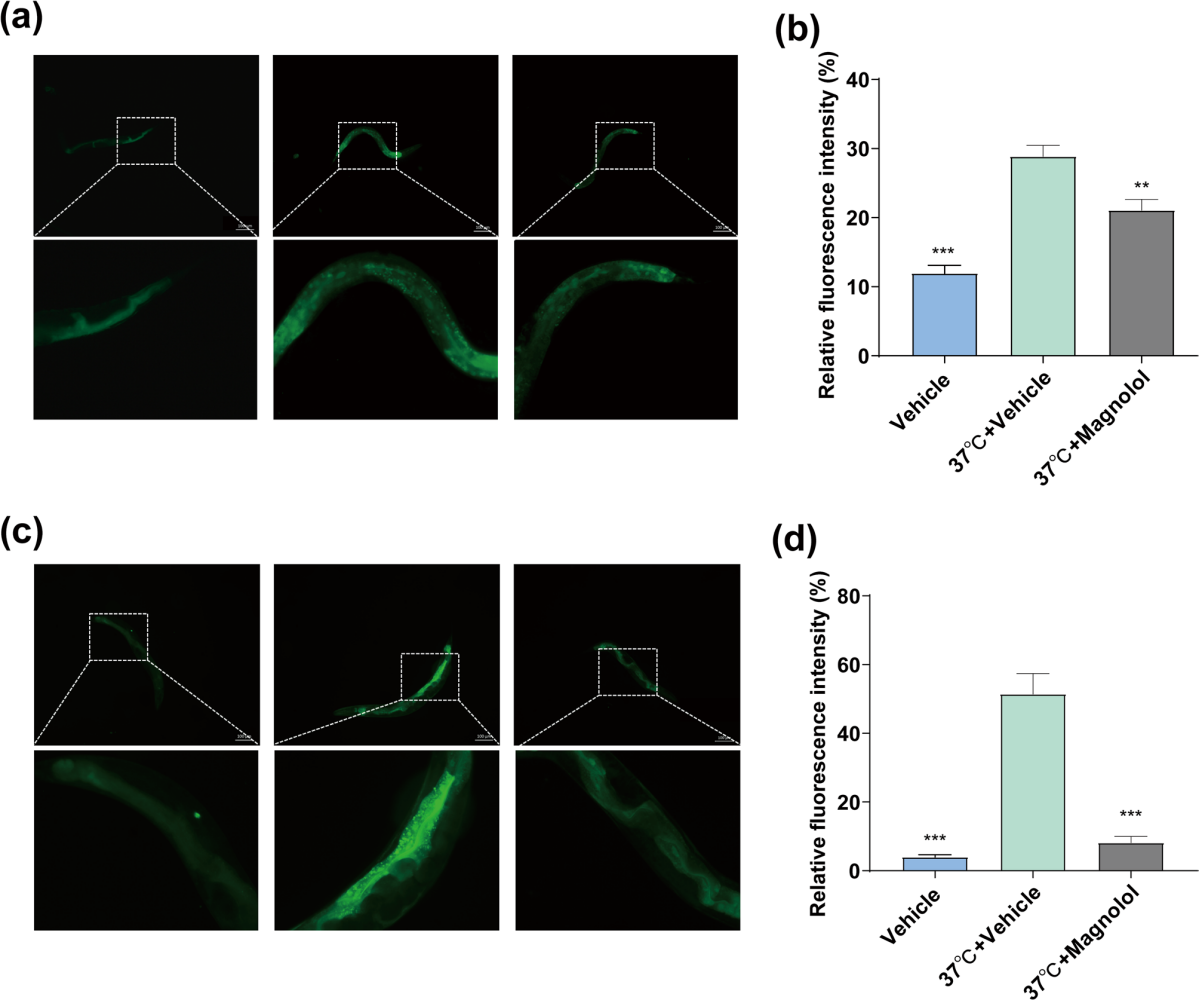
Magnolol decreases hsp-6 and hsp-16.2 under heat stress in *C. elegans*. (**a**) Fluorescence intensity images of HSP-16.2::GFP on the third day after treatment. (**b**) Statistics of the mean fluorescence intensity of HSP-16.2::GFP. (**c**) Fluorescence intensity images of HSP-6::GFP. (**d**) Statistics of the average fluorescence intensity of HSP-6::GFP. HSP-6::GFP*(zcIs13)* and HSP-16.2::GFP *(gpIs1)* worms were were treated with magnolol from Li to L3, then were placed on new NGM and incubated at 37 °C for 1 h. Data are expressed as mean ± SEM. n = 3 experiments, each involving 10 animals/group. **p < 0.01 and ***p < 0.001 compared to controls.

Protein homeostasis is another hallmarks of aging^48^. When the organism senses external stimuli, heat shock proteins can be induced to maintain protein homeostasis and increase the organism's ability to resist external stress^49^. Increase the functions of HSP-6 and HSP-16.2, the key regulators of mitochondrial and cytoplasmic unfolded proteins in nematodes, can improve stress resistance and prolongs lifespan of nematodes^50–52^. Here, *HSP-6::GFP(zcIs13)* and *HSP-16.2::GFP (gpIs1)* transgenic *C. elegans* were exposed in heat shock condition. Under heat stress conditions at 37 °C, the relative mean fluorescence intensity of HSP-6::GFP and HSP-16.2::GFP was significantly increased, whereas the magnolol treated worms attenuated the level of fluorescence intensity of HSP-6::GFP and HSP-16.2::GFP (Fig. 5a–d), suggesting that magnolol can increase the proteotoxic stress response of nematodes under heat stress conditions at 37 °C, which in turn maintains the protein homeostasis inside the organism to resist external stress and anti-aging.

### Lifespan-extending effect of magnolol is related to IIS signaling pathway

**Figure 6:**
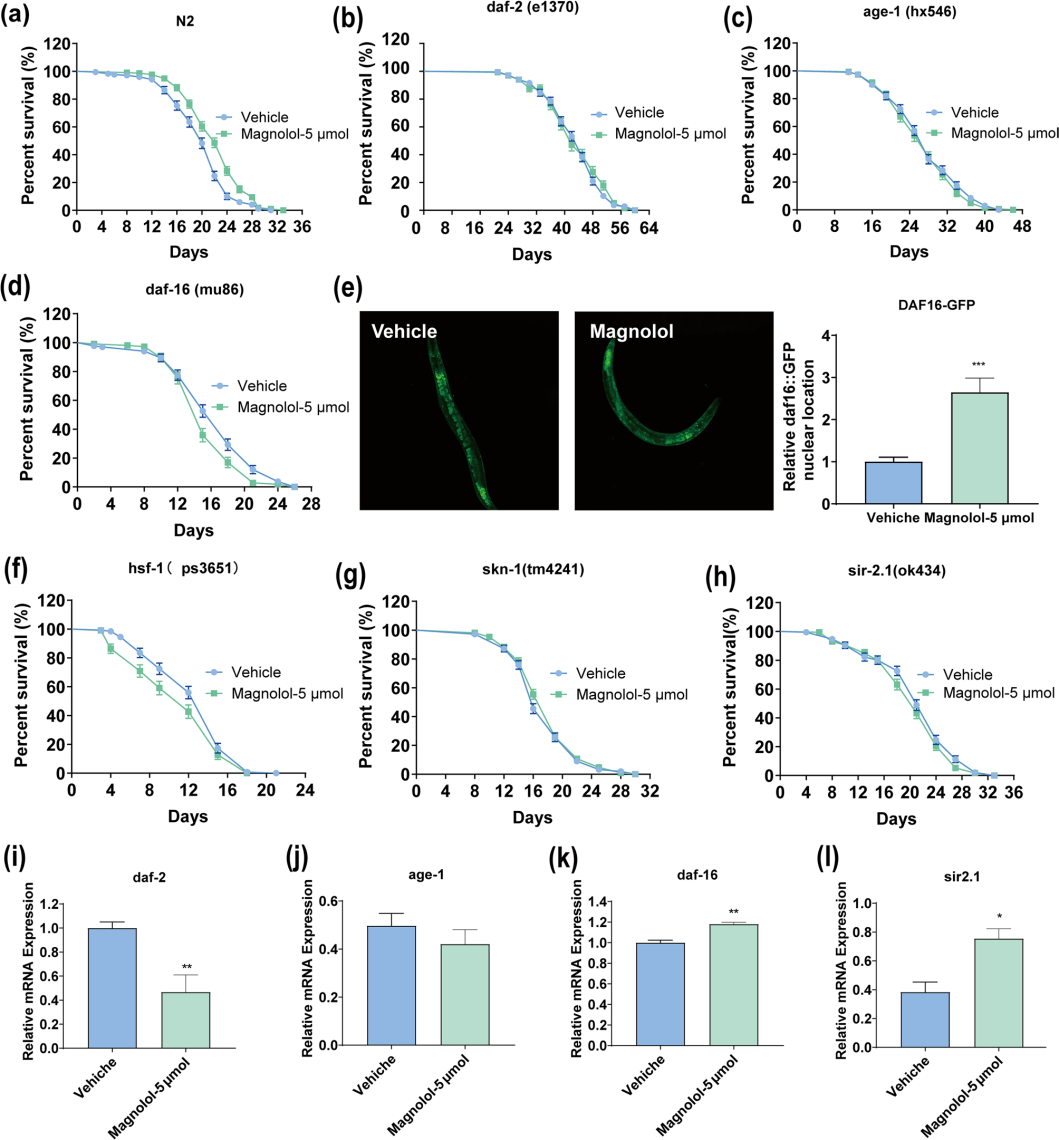
IIS pathway is involved in magnolol mediated lifespan extension. (**a**) The survival curve of wild type worms. (**b**) The survival curve of of *daf-2* (*e1370*). (**c**) The survival curve of *age-1* (*hx546*). (**d**) The survival curve of *daf-16* (*mu86*). (**e**) The nuclear translocalization of *DAF-16::GFP* (*muIs 109*) worms. At least 10 worms were analyzed. Scale bar, 100 μM. (**f**) The survival curve of *hsf-1* (*ps3651*) (**g**) The survival curve of *skn-1* (*tm4241*) (**h**) The survival curve of *sir2.1* (*ok434*). (**i**) The expression of the downstream genes of daf-16, including age-1, daf-2 and sir 2.1 tested by qRT-PCR. n = 106–146 nematodes for each group (**a**–**d**, **f**–**h**). Lifespans were performed at 20 °C, and analyzed by log-rank test. The data graphed by GraphPad, and the SPSS was used to calculate the significant differences. The data were expressed as mean lifespan ± S.E.M. The detailed lifespan data were exhibited in Table 4,*p < 0.05, **p < 0.01, ***p < 0.001.

The insulin/insulin-like growth factor signaling pathway (IIS) plays an important role in nematode tolerance to various stressors and longevity^17,53^. To investigate the mechanism of longevity extension by magnolol, we tested the lifespan of key regulators in IIS pathway, *daf-2*, *age-1* (mammalian PI3K homolog) and *daf-16* (mammalian FOXO) mutants. We found that magnolol failed to prolong the lifespan of *daf-16* (*mu86*), *daf-2* (*e1370*) and *age-1* (*hx546*) mutant nematodes compared to N2 (Fig. 6a–d, Table 4). In addition, nuclear translocation of DAF-16 was significantly promoted by magnolol treatment as visualized by GFP-fluorescence in a DAF-16::GFP transgenic strain (Fig. 6e). Moreover, the mRNA expression levels of *daf-2* was significantly reduced, while *daf-16* was significantly up-regulated in N2 after magnolol treatment (Fig. 6i–l).

**Table 4:** Effects of magnolol on the lifespan of N2, *daf-2*, *age-1*, *daf-16*, *skn-1*, *hsf-1* and *sir2.1 C. elegans.*

Strain	Group	Number	Mean Survival ± SEM (hours)	Maximum longevity (days)	Median survival time (days)	Increase (%)	*p* value
N2	Vehicle	170	20.012 ± .372	31	20	/	
	Magnolol-5 μM	212	22.292 ± 0.313	33	23	11.39	< 0.001***
*daf-2*	Vehicle	214	43.164 ± 0.553	57	45	/	/
	Magnolol-5 μM	134	43.276 ± 0.748	57	42	− 0.26	0.545
*age-1*	Vehicle	200	27.770 ± 0.525	40	28	/	/
	Magnolol-5 μM	146	27.185 ± 0.583	43	28	− 2.11	0.306
*daf-16*	Vehicle	133	16.714 ± 0.403	26	18	/	/
	Magnolol-5 μM	106	15.632 ± 0.437	26	15	− 6.47	0.012
*skn-1*	Vehicle	211	17.679 ± 0.301	28	16	/	/
	Magnolol-5 μM	213	18.123 ± 0.305	28	19	2.51	0.908
*hsf-1*	Vehicle	127	12.929 ± 0.357	21	15	/	/
	Magnolol-5 μM	110	11.432 ± 0.437	18	12	− 11.57	0.026
*sir2.1*	Vehicle	186	21.419 ± 0.458	30	21	/	/
	Magnolol-5 μM	171	20.684 ± 0.4452	30	21	− 3.43	0.105

Lifespan experiments were analysed using Kaplan–Meier survival analysis and compared among groups, scoring for significance using the log-rank test. All data were expressed as mean ± SEM. ***p < 0.001 versus control group.

Contemporary studies have shown that *hsf-1*, a heat stress regulator, and *skn-1*, the homologous gene of Nrf2, are downstream targets of insulin-like signaling pathway *daf-16*^54,55^. So we conducted longevity assays on *skn-1* (*tm4241*) and *hsf-1* (*ps3651*) mutants. Interestingly, we found that similar to in *daf-16* (*mu86*), *daf-2* (*e1370*) and *age-1* (*hx546*), there was no response of lifespan in *skn-1* (*tm4241*) and hsf-1 (*ps3651*) with magnolol treatment (Fig. 6f, g, Table 4). RT-PCR analysis showed that magnolol was able to up-regulate *sod-3*, *mtl-1, cat-1* and *hsp-16.2,* which are oxidative stress-related genes (Fig. 3p).

It has been reported that the nematode *sir-2.1* gene, which is homologous to the human *sirtuin 1* (*SIRT1*), can control cellular response to stress by directly or indirectly regulating the activity of daf-16/FOXO through deacetylation. In addition, in cells, SIRT1 has a bidirectional regulator of FOXO, and this two-sided regulation contributes to the increase of cell survival, which prolongs the lifespan of organism and slows down aging process. Indeed, when *sir-2.1* (*ok434*) nematode were treated with magnolol, there were a similar mean and maximal survival rates as vehicle treatment (Fig. 6h, Table 4). The result suggests that magnolol may increase the stress resistance of nematodes by regulating *sir-2.1*, which in turn regulates the IIS signaling pathway, and ultimately affects the lifespan of nematodes.

### The effects of magnolol on aging-related neurodegenerative diseases

**Figure 7:**
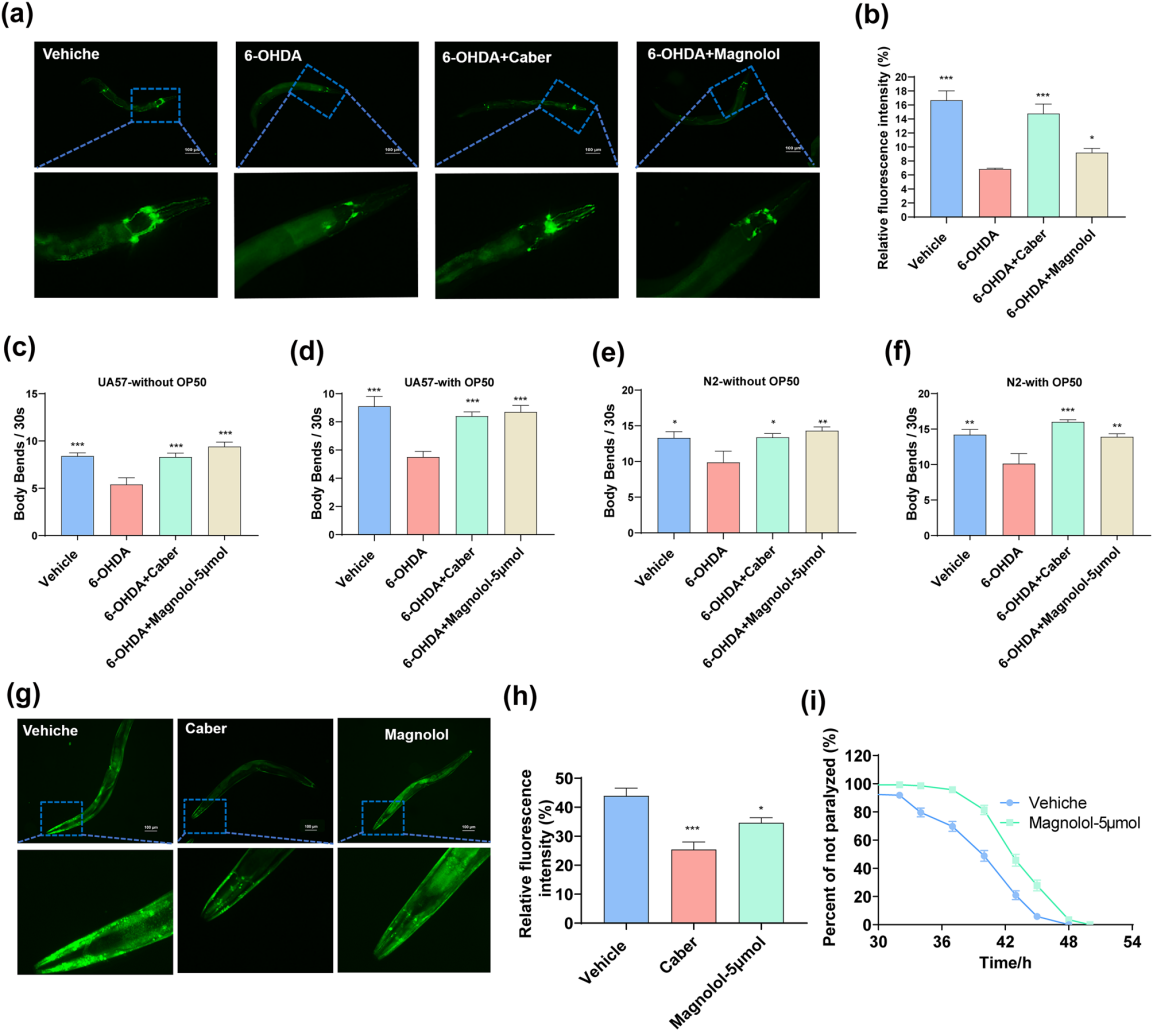
Therapeutic effects of magnolol on aging-related neurodegeneration. (**a**, **b**) Magnolol ameliorates dopamine neuron degeneration in nematode *UA57*. (**c**, **d**) Magnolol ameliorates 6-OHDA-induced nematode strain N2 and UA57 locomotion deficits. (**e**, **f**) Effect of magnolol on 6-OHDA-induced locomotion of wild-type nematode N2. (**g**, **h**) Magnolol ameliorates the accumulation of α-synuclein in the muscle tissue of the body wall of nematode *NL5901*. Positive control groups were treated with cabergoline (Caeber). (**i**) Paralysis of *CL4176* induced by Aβ. All data were presented as mean ± S.E.M. The detailed lifespan data were exhibited in Table 5. Compared with vehicle group, *p < 0.05, **p < 0.01, ***p < 0.001.

Considering the promising anti-aging activity of magnolol, we further explored its roles in Parkinson’s disease (PD) and Alzheimer’s disease (AD) models of *C. elegans*. Age-related loss of dopaminergic neurons is an important feature of PD. There are 8 dopaminergic neurons in nematodes, including 6 in the head (4 CEP nerves and 2 ADE neurons) and 2 in the tail (PDE neurons). UA57 (*pkls2386*) is a classic strain for PD studies marked dopaminergic neurons with GFP. Here, we treated UA57 worms with 6-OHDA to induce a degeneration of dopaminergic neurons^56^. The results showed that the fluorescence intensity was increased in the magnolol-treated group compared to that in the control (Fig. 7a, b). In addition, by observing the body bends of nematodes, it was found that magnolol could improve the limb incoordination of N2 and UA57 caused by 6-OHDA (Fig. 7c–f). It suggests that magnolol can prevent the damage to dopaminergic neurons caused by the neurotoxic substance 6-OHDA to a certain extent, and play a neuroprotective role in pathological conditions. Pathological α-synuclein aggregation has been reported to be associated with the decline of dopaminergic neurons^57^. To further confirm the effects of magnolol on PD, we studied NL5901 (*bals4*) worm, a transgenic model of PD created by inserting the human *α-synuclein* gene^56^. It was found that the magnolol-treatment attenuated pathological α-synuclein aggregation compared to the control group (Fig. 7g, h).

In AD brain, one of the most obvious clinical pathologic features is amyloid (A-β, Aβ-Protein) deposition^58^. We found that in the nematode strain *CL4176*, a human A-β was induced in muscle cells when induced at 25 °C, magnolol treatment significantly prolonged the time to onset of paralysis compared to the control group. This suggests that magnolol may inhibit the toxic effects of A-β in worms (Fig. 7i, Table 5). In summary, magnolol may have neuroprotective effects on age-related neurodegenerative diseases.

**Table 5:** Effects of magnolol on paralysis of *CL4176* induced by Aβ.

Strain	Group	Paralysis number	Mean paralysis-free time ± SEM (hours)	Maximum paralysis-free time (hours)	Median paralysis-free time (hours)	Increase (%)	*p* value
*CL4176*	Vehicle	172	40.378 ± 0.345	48	40	/	/
	Magnolol-5 μM	140	44.079 ± 0.283	50	43	9.17	< 0.001***

Survival analysis of paralysis were used Kaplan–Meier survival analysis and compared among groups, scoring for significance using the log-rank test. All data were expressed as mean ± SEM. ***p < 0.001 versus control group.

Then, the resistance-related gene *skn-1* were knocked down using RNAi in NL5901 and CL4176 worms and treated with magnolol. The results showed that the effects of magnolol on pathological α-synuclein aggregation and paralysis were attenuated in *skn-1* deficient worms (Fig. S1d–f, Supplementary Table S1) when compared to those of worms treated with an empty control vector (L4440) (Fig. S1a–c). Collectively, these data indicate that magnolol can improve age-related neuropathological changes through the stress resistance gene *skn-1*.

## Discussion

In recent years, the anti-aging properties of natural products have attracted much attention because of less side effects and toxicity^59^. However, aging is a complex process and the lack of scientific evidence in most cases to demonstrate the exact underlying molecular mechanisms, which limits the application of such natural products in anti-aging practice. Magnolol has been used in traditional medicine as a time-honored mild herb with many beneficial clinical properties^27,60^. More notably, magnolol has been reported in literature to alleviate UV-induced photoaging effects in hairless mouse skin^61^, improve motor and memory abilities in various aging models such as SAMP8 mice^62^, and prolong the lifespan of wild-type *Saccharomyces cerevisiae*^63^. However, research on the role of magnolol in prolonging the aging and its mechanisms are still incomplete.

In the present study, magnolol increased the average lifespan of N2 *C. elegans* by 13.14% (Fig. 1a, Table 2), as well as significantly improved pharyngeal pumping and body flexion in aged nematodes (Fig. 1d–g), inhibited lipid accumulation (Fig. 2a, b) and mitochondrial integrity (Fig. 4a–d). Our study provides the first more comprehensive account of the life-extending effects of magnolol in terms of both lifespan and health span extension. A restricted diet is known to be the most powerful method for extending the lifespan of nematodes. We found that magnolol did not affect the growth of OP50 (Fig. 1b), and the nematodes showed no preference for the foods (Fig. 1c, d). Thus, the lifespan-extending effect of magnolol is not related to dietary restriction. In addition, magnolol had no effect on the average number of hatchlings and larval offspring of nematodes. Taken together, magnolol may be a safe anti-aging ingredient.

Magnolol has been reported to protect neuronal damage in MPTP-induced Parkinson's disease mice^64^, and to ameliorate cognitive deficits in TgCRND8 transgenic mice and APP/PS1 mice^65,66^. Not only that, magnolol also can prevent age-related learning and memory impairments by protecting cholinergic neurons in the forebrain^62^. Consistent with these results, our results showed that magnolol delayed the rate of paralysis in AD model *CL4176* nematode (Fig. 7i, Table 5). In addition, magnolol prevented the damage of the neurotoxic substance 6-OHDA to dopaminergic neurons in UA57 nematode (Fig. 7a–f), and improved the protein homeostasis in NL5901 model by decreasing the accumulation of α-synuclein (Fig. 7g, h), which exerts neuroprotective effects under pathological conditions.

Various stressors in the environment are risk factors for human health and are associated with many age-related diseases, such as Alzheimer's disease and Parkinson's disease^67–70^. Increased stress resistance is a common feature of long-lived worms, flies and rodents, suggesting that stress resistance may be associated with longevity^71^. Magnolol has been shown to have significant anti-oxidative stress effects^72^. Existing studies have shown that magnolol can protect yeast antioxidant gene-deficient mutant strains from oxidative stress^60^, enhance the expression of antioxidant enzymes such as SOD, CAT and reduce the levels of oxidized products ROS and MDA in a variety of senescence models^61,62^. These studies suggest that the anti-aging effects of magnolol may be related to increasing the body's ability to combat oxidative stress, however, the underlying mechanisms of effect are not clear. Similarly, in the present study, magnolol increased nematode survival under H_2_O_2_ stress conditions, decreased the levels of oxidation products ROS and MDA, and enhanced the resistance to oxidative stress in N2 (Fig. 3b, k–n). In addition, we also found that magnolol increased the survival rate of nematodes under heat stress at 37 °C, decreased the fluorescence intensity of HSP-6::GFP under heat stress (Figs. 3a and 5c, d), suggesting that the enhancement of the ability to resist heat stress may be a new mechanism for the anti-aging effect of magnolol.

When the organism is exposed to external stimuli, the enzymes that rapidly metabolize toxic chemicals (e.g., cytochrome P450) are activated in addition to the stress response pathway to increase tolerance to the toxicants. In our study, we found for the first time that the increase in stress tolerance of magnolol is closely related to the increase in detoxification capacity. In our study, moderate amount of magnolol increased nematode survival when exposed to PQ, MeHgCl and colchicine, and increased cellular activity of HepG2 and L02 exposed to both PQ and MeHgCl. Meanwhile, magnolol up-regulated the expression of genes related to detoxification enzymes in nematodes, such as *cyp13a4*, *cyp14a1*, *cyp14a3, cyp36a1*, *cyp35b2*, *cyp35b3*, *gst-4*, *gst-10* and *pgp-13* (Fig. 3c–j). These results suggest that the role of magnolol in prolonging nematode lifespan may be related to enhancement of stress resistance.

It has been well established that IIS pathway is closely related to nematode senescence and stress tolerance, and that *skn-1* and *hsf-1* are key downstream targets of insulin signaling pathway that regulate heat shock response, oxidative stress and detoxification capacity of nematodes. In the present study, magnolol failed to prolong the lifespan of *daf-2*, *age-1*, *daf-16*, *hsf-1* and *skn-1* mutant lines (Fig. 6a–d, f, g), while magnolol decreased the expression of *daf-2* genes (Fig. 6i) and up-regulated the expression of *daf-16* downstream genes of *hsf-1* and *skn-1*, such as *sod-3*, *cat-1*, *mtl-1* and *hsp-16.2* (Fig. 3p), facilitated the transfer of *daf-16* from cytoplasm to nucleus (Fig. 6e) and decrease the fluorescence intensity of HSP-16.2::GFP under heat shock (Fig. 5a, b). These results suggest that the prolonged lifespan effect of magnolol is achieved by inhibiting IIS signaling pathway. Another interesting occurrence in our experiments was the absence of lifespan-extending effects of magnolol on *sir-2.1* mutant nematodes (Fig. 6h), a key factor in the regulation of the activity of *daf-16*/FOXO, suggesting that *sir-2.1/*SIRT1 may be a key target of magnolol anti-aging. The overall data suggest that the mechanism of magnolol on lifespan-extension in *C. elegans* may be related to the increase in stress resistance mediated by IIS signaling pathway.

Neurological damage is closely associated with increased oxidative stress^73^. Alleviation of oxidative stress has been shown to inhibit Aβ and α-synuclein aggregation^52,74^. Our study found that magnolol has a significant anti-inflammatory effect. In addition, we also found that the neuroprotective effects of magnolol on both AD and PD neuroprotection in aging-related diseases are closely related to the anti-stress gene *skn-1* using RNAi interference experiments (Fig. S1d–f, Supplementary Table S1). However, whether *skn-1* can promote the clearance of Aβ and α-synuclein aggregates is needed further investigation. Recently, it has been reported in literature that up-regulated expression of heat shock proteins promotes normal protein folding and degrades misfolded cytoplasmic proteins, thereby helping to achieve protein homeostasis^41^. Magnolol had a significant regulatory effect on the expression of both *hsp-6* and *hsp-16.2*. However, it remains to be further verified whether the effects of magnolol on lifespan-extension and neurological diseases are related to protein homeostasis.

In conclusion, we found that magnolol can prolong the lifespan and health span of *C. elegans*, and the lifespan-extending effect of magnolol is achieved by modulating IIS signaling pathway to increase the resistance to oxidative stress, heat stress and toxicants. Our study provides a novel mechanism for the anti-aging effects of magnolol.

### Supplementary Information

Supplementary Information.

## Data Availability

All data generated or analyzed during this study are included in this published article.
