# Supplementary material for: Magnolol extends lifespan and improves age-related neurodegeneration in Caenorhabditis elegans via increase of stress resistance
Source: Sci Rep. 2024 Feb 7;14:3158. doi: 10.1038/s41598-024-53374-9 (PMC10850488; doi:10.1038/s41598-024-53374-9)
Supplement: Supplementary file 1 — Supplementary Information. [file 41598_2024_53374_MOESM1_ESM.docx]

Supplementary Information for

## Magnolol extends lifespan and improves age-related neurodegeneration in *C. elegans* via increase of stress resistance

Jing Yu^1, 2^, Xiaoyan Gao^1, 2^, Lijun Zhang^1^, Hang Shi^1^, Yingxuan Yan^1^, Yongli Han^1^, Chengyuan Wu^1^, Ying liu^1^, Minglv Fang^1^, Cheng Huang^1^*, Shengjie Fan^1^*

Affiliations:

1 School of Pharmacy, Shanghai University of Traditional Chinese Medicine, Shanghai, 201203, China

2 These authors contributed equally: Jing Yu, Xiaoyan Gao

*Correspondence: Cheng Huang: chuang_shutcm@163.com; or Shengjie Fan: shengjiefan@shutcm.edu.cn

This Word file includes: Supplementary Figures 1

Supplementary Tables 1

Supplementary Methods

Supplementary Results

Supplementary Fig. S1


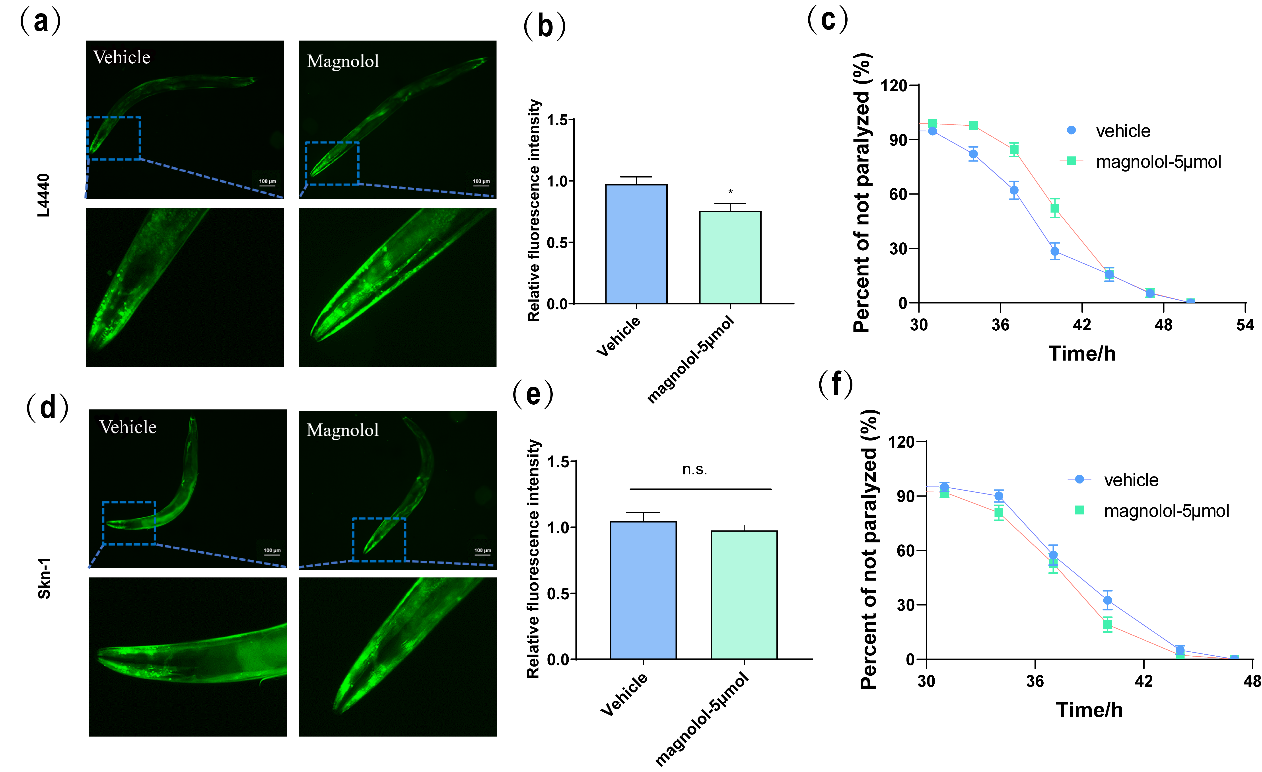


**Fig. S1.** **Magnolol alleviates AD and PD-like symptoms of worms through SKN-1 signal pathway**. (a-b) Magnolol ameliorated the accumulation of α-synuclein in the muscle tissue in *NL5901* worms without *skn-1* RNAi. (d-e) Magnolol failed to ameliorate the accumulation of α-synuclein in the muscle tissue in *NL5901* worms with *skn-1* RNAi. (c) Curves of not paralyzed fraction in *CL4176* worms after treatment without *skn-1* RNAi. (f) Curves of not paralyzed fraction in *CL4176* worms after treatment with *skn-1* RNAi. All data were presented as mean ± S.E.M. The detailed lifespan data were exhibited in Supplementary Table S1. Compared with vehicle group, * p <0.05, ** p < 0.01.

**Supplementary Table S1.** Effects of magnolol on paralysis of *CL4176* induced by Aβ with or without skn-1 RNAi

| Strains and RNAi | Group | Paralysis number | Mean paralysis-free time± SEM （hours） | Maximum  paralysis-free time (hours) | Median  paralysis-free time  (hours) | Increase （%） | p value |
| --- | --- | --- | --- | --- | --- | --- | --- |
| L4440 | Vehicle | 95 | 39.937±0.495 | 50 | 40 | / | / |
|  | Magnolol-5 μM | 90 | 42.156±0.399 | 50 | 44 | 5.56 | ＜0.01** |
| *Skn-1* | Vehicle | 80 | 39.725±0.444 | 47 | 40 | / | / |
|  | Magnolol-5 μM | 89 | 38.607±0.410 | 47 | 40 | -2.81 | 0.063 |

Notes: Survival analysis of paralysis were used Kaplan-Meier survival analysis and compared among groups, scoring for significance using the log-rank test. All data were expressed as mean± SEM. **p < 0.01 vs. control group

**Supplementary Methods**

The analysis of *skn-1* RNA interference (RNAi) was performed as described previously^35^. Briefly, RNAi bacterias were grown at 37°C in LB liquid medium overnight with 50 μg/ml ampicillin, and then seeded onto NGM plate containing 50 μg/ml ampicillin and 1 mg/ml isopropylthiogalactoside (IPTG), cultured overnight. L1 stage NL5901 and CL4176 worms were transferred onto the plate with RNAi bacteria lawn until L4 stage. The remaining steps were carried out as described in the above.

**Supplementary Results**

Then, the resistance-related gene *skn-1* were knocked down using RNAi in NL5901 and CL4176 worms and treated with magnolol. The results showed that the effects of magnolol on pathological α-synuclein aggregation and paralysis were attenuated in *skn-1* deficient worms (Fig. S1 d, e, f, Supplementary Table S1) when compared to those of worms treated with an empty control vector (L4440) (Fig. S1 a, b, c). Collectively, these data indicate that magnolol can improve age-related neuropathological changes through the stress resistance gene *skn-1*.
